# Isolation and Functional Characterization of an Acidic Myotoxic Phospholipase A_2_ from Colombian *Bothrops asper* Venom

**DOI:** 10.3390/toxins9110342

**Published:** 2017-10-26

**Authors:** Silvia Posada Arias, Paola Rey-Suárez, Andrés Pereáñez J, Cristian Acosta, Mauricio Rojas, Lucilene Delazari dos Santos, Rui Seabra Ferreira Jr, Vitelbina Núñez

**Affiliations:** 1 Programa de Ofidismo y Escorpionismo, Universidad de Antioquia, Medellín 050010, Colombia; ofidpa@gmail.com (P.R.-S.); andrespj20@gmail.com (A.P.J.); cj.acosta1@hotmail.com (C.A.); vitelbina.nunez@gmail.com (V.N.); 2 Research Group in Veterinary Medicine GIVET, School of Veterinary Medicine, Corporación Universitaria Lasallista, Caldas-Antioquia 055440, Colombia; 3 Grupo de Inmunología Celular e Inmunogenética (GICIG), Universidad de Antioquia, Medellín 050010, Colombia; mauricio.rojas@udea.edu.co; 4 Center for the Study of Venoms and Venomous Animals (CEVAP), Universidad Estadual Paulista (UNESP) and Graduate Program in Tropical Diseases, Botucatu Medical School (FMB), Botucatu, São Paulo 18610-307, Brazil; lucilenebio@gmail.com (L.D.d.S.); rui.ead@gmail.com (R.S.F.J.); 5 Escuela de Microbiología, Universidad de Antioquia, Medellín 050010, Colombia

**Keywords:** acidic myotoxic phospholipase A_2_, *Bothrops asper*, edema, myotoxicity, snake venom

## Abstract

Myotoxic phospholipases A_2_ (PLA_2_) are responsible for many clinical manifestations in envenomation by *Bothrops* snakes. A new myotoxic acidic Asp49 PLA_2_ (BaCol PLA_2_) was isolated from Colombian *Bothrops asper* venom using reverse-phase high performance liquid chromatography (RP-HPLC). BaCol PLA_2_ had a molecular mass of 14,180.69 Da (by mass spectrometry) and an isoelectric point of 4.4. The complete amino acid sequence was obtained by cDNA cloning (GenBank accession No. MF319968) and revealed a mature product of 124 amino acids with Asp at position 49. BaCol PLA_2_ showed structural homology with other acidic PLA_2_ isolated from *Bothrops* venoms, including a non-myotoxic PLA_2_ from Costa Rican *B. asper*. In vitro studies showed cell membrane damage without exposure of phosphatidylserine, an early apoptosis hallmark. BaCol PLA_2_ had high indirect hemolytic activity and moderate anticoagulant action. In mice, BaCol PLA_2_ caused marked edema and myotoxicity, the latter seen as an increase in plasma creatine kinase and histological damage to gastrocnemius muscle fibers that included vacuolization and hyalinization necrosis of the sarcoplasm.

## 1. Introduction

Colombia has the third highest biodiversity of snakes in the Americas [1]. In 2015, there were 4273 clinically confirmed cases of snakebite in the country, with an average of 82.2 snakebites per week and an incidence of 8.84 cases per 100,000 habitants [2]. Approximately 94.6% of the snakebites are caused by snakes of the genus *Bothrops* [2].

In Colombia, *Bothrops asper* is found in the Pacific, Caribbean and Andean (western slopes) regions where it accounts for 50–80% of snakebites [2]. Envenomation by *B. asper* leads to sequelae in 6–10% of cases [2,3], with the most important being the loss of muscle mass leading to limb dysfunction or amputation as a result of myonecrosis, dermonecrosis and severe edema that induces ischemia [3]. The main toxins implicated in these effects are snake venom metalloproteinases (SVMPs) and PLA_2_s [4,5]. Proteomic analyses have shown that *B. asper* venom contains at least seven protein families including disintegrin, phospholipases A_2_, serine proteinases, *C*-type lectins, cysteine-rich secretory proteins (CRISP), *L*-amino acid oxidase, and Zn^2+^-dependent metalloproteinases of which PLA_2_ are an important group [6]. PLA_2_, which hydrolyze phospholipids at the *sn*-2 position to release fatty acids and form lysophospholipids [7], can cause myotoxicity, edema, neurotoxicity, cardiotoxicity and hemolysis, in addition to affecting coagulation, platelet aggregation and showing antibacterial activity [8].

Based on the amino acid sequence and pattern of disulfide bonds, snake venom PLA_2_ have been classified in two groups: Group I PLA_2_ found in venoms of the Elapidae and Hydrophidae and Group II PLA_2_ present in the family Viperidae [9]. PLA_2_s of the latter group are divided into two subgroups: Asp49 PLA_2_s that include catalytically active isoforms and Lys49 PLA_2_s devoid of enzymatic activity [4]. Asp49 PLA_2_s display a wide range of isoelectric points, from acidic to basic, with the acidic isoforms usually having greater catalytic activity but less toxicity than basic PLA_2_s [10,11]. Snake venom acidic PLA_2_s have not been studied as extensively as other PLA_2_s and many of those that have been characterized have been described as non-myotoxic and are involved in pharmacological effects such as hypotension and the inhibition of platelet aggregation [12]. In this paper, we describe the isolation and characterization of a myotoxic acidic PLA_2_ (BaCol PLA_2_) from *B. asper* venom.

## 2. Results

### 2.1. Isolation, Determination of Molecular Mass, Sequencing and Modeling of BaCol PLA_2_

**Figure 1:**
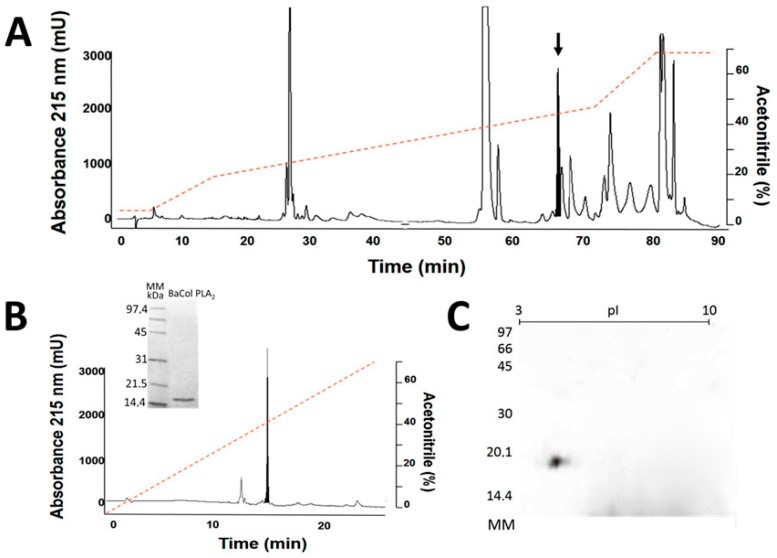
Isolation of BaCol PLA_2_ (acidic Asp49 phospholipases A_2_). (**A**) Elution profile of *B. asper* venom by RP-HPLC (reverse-phase high performance liquid chromatography) on a Resteck C18 semi-preparative column. The fraction indicated by the arrow showed high PLA_2_ activity; (**B**) The PLA_2_ fraction was analyzed by RP-HPLC and purity was assessed by SDS-PAGE on a 12% polyacrylamide gel in reducing conditions. MM–molecular mass markers (in kDa); (**C**) Isoelectric focusing in a 10% polyacrylamide gel (pI range: 3–10). MM: molecular mass markers (in kDa).

**Figure 2:**
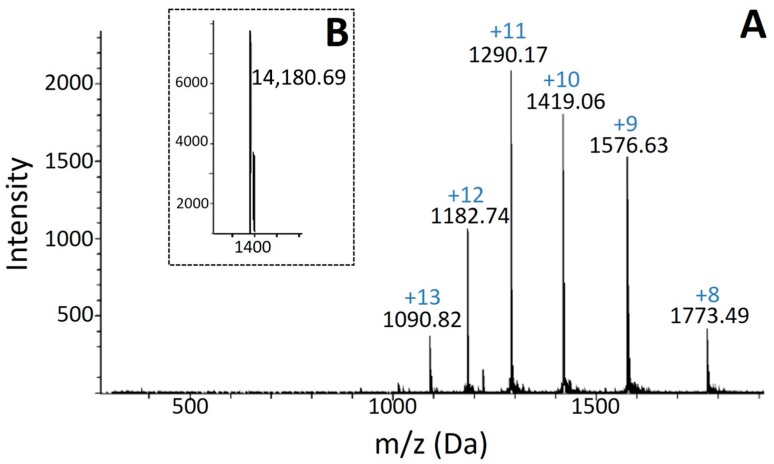
Mass spectrometric analysis of BaCol PLA_2_. (**A**) Spectrum obtained in multi-charge mode, as described in Materials and Methods; the inset (**B**) shows the deconvolution of the multi-charged ion series indicated in (**A**).

Fractionation of *B. asper* venom by RP-HPLC resulted in 16 major peaks (Figure 1A) that were collected and screened for PLA_2_ activity. Peak 6 (retention time: 67.48 min) showed high PLA_2_ activity. Analytical chromatography of this peak by RP-HPLC resulted in a single symmetric peak. SDS-PAGE of the purified protein under reducing conditions showed a single band migrating at ~14.5 kDa (Figure 1B) with an isoelectric point (pI) of 4.4 based on isoelectric focusing (Figure 1C). The molecular mass obtained by ESI-Q-ToF was 14,180.69 Da (Figure 2A,B). This protein was named BaCol PLA_2_.

**Figure 3:**
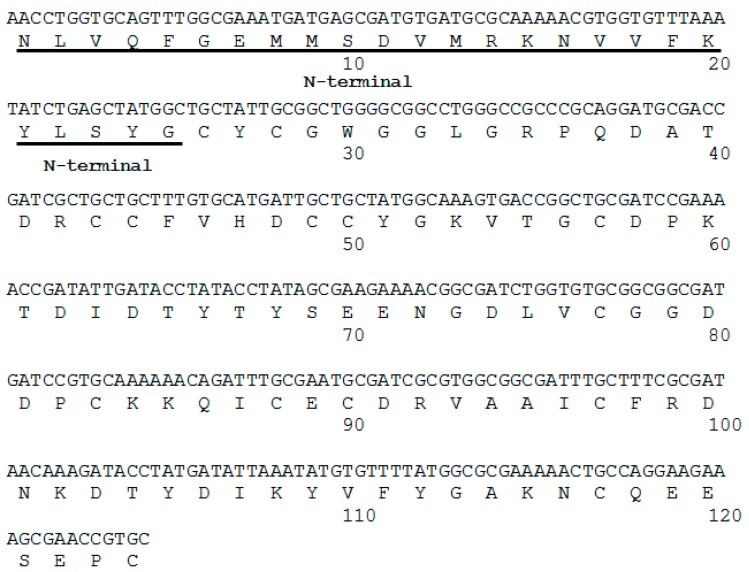
cDNA and translated amino acid sequence of BaCol PLA_2_. The *N*-terminal sequence (first 25 amino acids) determined by direct Edman degradation is underlined.

The first 25 amino acids of the *N*-terminal region were determined by Edman degradation. This sequence was used to design the primers to obtain the corresponding cDNA from *B. asper* venom gland mRNA. The cDNA encoded a polypeptide 124 amino acids long (Figure 3), with the presence of Asp at position 49 of the catalytic dyad (based on the numbering of Renetseder et al. [13]) and a theoretical pI of 4.5. The *N*-terminal sequence obtained by Edman degradation was identical to that deduced from cDNA (Figure 3). The DNA sequence was deposited under accession number MF319968.

**Figure 4:**
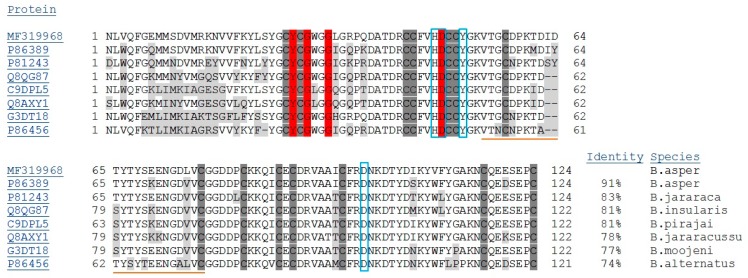
Multiple sequence alignment of BaCol PLA_2_ with other PLA_2_ isolated from *Bothrops* snake venoms. Protein access codes are indicated in the first column. The number of amino acids, percentage identity compared to BaCol PLA_2_ and the species of origin are also indicated. Cysteine residues are highlighted in dark gray and amino acid sequence variants in light gray. The loop for calcium residues are highlighted in red. The residues of the active site are indicated with blue boxes. The residues of the putative site for anti-clotting activity are underlined with orange.

Alignment of the amino acid sequence with other PLA_2_s is shown in Figure 4. There was a high percentage of identity with a Costa Rican *B. asper* PLA_2_ (BaPLA_2_-II; 91%) and with a PLA_2_ from *B. jararaca* (BJPLA_2_; 83%), both of which have 124 amino acids. There was 81% identity with BinTX-I, a 138-amino acid (including signal peptide) PLA_2_ from *B. insularis*, and BpirPLA_2_-I, a 122-amino acid PLA_2_ from *B. pirajai*. Lower identity was observed with PLA_2_ from *B. jararacussu* (Bth-A-I-PLA_2_), *B. moojeni* (Bmoo-PLA_2_) and *B. alternatus* (Balt1) (78%, 77% and 74%, respectively), all of which are acidic PLA_2_.

**Figure 5:**
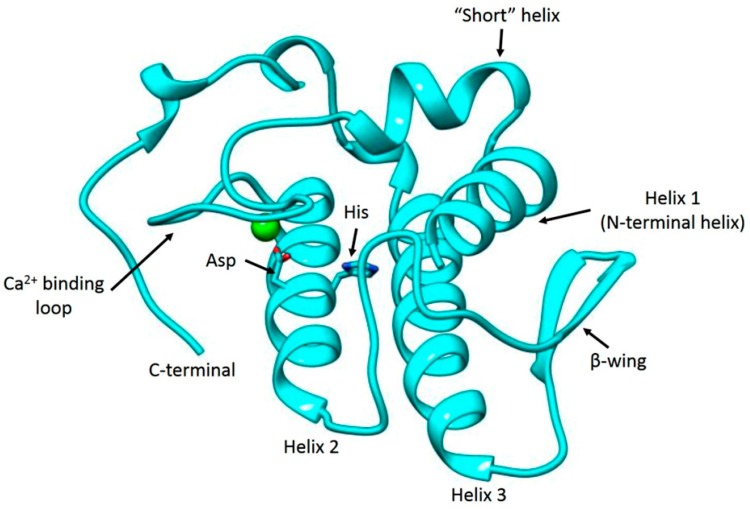
Three-dimensional model of BaCol PLA_2_. The active site residues (Asp and His) of the enzyme are shown in sticks and Ca^2+^ is shown as a light green sphere.

After National Center for Biotechnology Information Basic Local Alignment Search Tool (NCBI BLAST) the chain *X* of PLA_2_ from *Bothrops jararacussu* (PDB ID: 1UMV_X) was chosen as template for our homology modeling process. The template has a resolution of 1.79 Å, an identity score of 78%, an *E* value of 7 × 10^−67^ and coverage of 100% with BaCol PLA_2_. Protein modeling yielded a 3D-structure with the general characteristics of venom PLA_2_s, i.e., a calcium-binding loop, two antiparallel helixes, a *β*-wing and a *C*-terminal loop (Figure 5, Supplementary Materials Figure S1). The reliability and quality of the structural model was assessed by Procheck. The detailed residue-by-residue stereochemical quality of the BaCol PLA_2_ model was found to be good 91.5% in the most favored regions, 7.5% in additional allowed regions, 1% in generously allowed regions (Arg14) and 0% in disallowed regions (Supplementary Materials Figure S2).

The Verify 3D program was used to determine the compatibility of an atomic model (3D) with its own amino acid sequence (1D). This software considers a good score when at least 80% of the amino acids score ≥0.2 in the 3D/1D profile; for BaCol PLA_2_, this value was 87.1%, with scores ranging from 0.05 to 0.53 (Supplementary Materials Figure S3 and Table S1). The energetic architecture of the protein folds was determined with the program ProSA and yielded a *Z*-score of −5.2, which is within the range of native conformations for the template (−4.49) (data not shown). The energy profile of the BaCol PLA_2_ predicted model was thus found to be good (Supplementary Materials Figure S4).

### 2.2. Biological Activities

**Figure 6:**
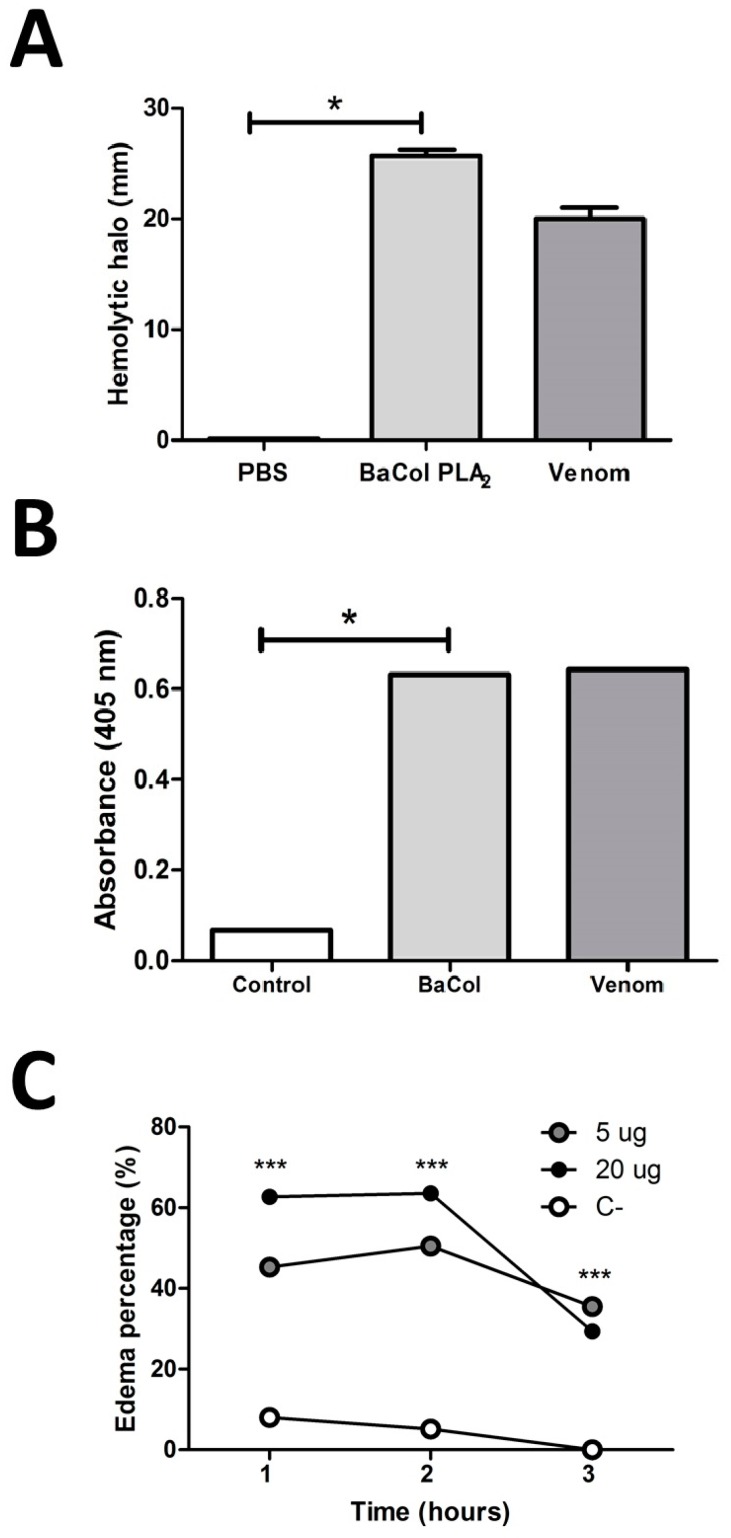
BaCol PLA_2_ activity and edema formation. (**A**) Indirect hemolytic activity of BaCol PLA_2_ (15 µg/µL) and venom (15 µg/µL) assayed using human erythrocytes and egg yolk as substrate. Activity was expressed as the diameter of the hemolytic halo after incubation for 20 h at 37 °C (**B**) Hydrolysis of the synthetic substrate 4-nitro-3-octanoyloxy-benzoic acid by BaCol PLA_2_ (1 µg/µL) and venom (1 µg/µL), measured as the increase in absorbance after incubation for 1 h at 37 °C; (**C**) Mouse hind paw edema induced by BaCol PLA_2_. PBS (50 µL) (white circles) or BaCol PLA_2_ (5 µg—gray circles or 20 µg—black circles) in 50 µL of PBS was injected into the hind footpad of male Swiss-Webster mice and edema formation was monitored as the increase in footpad thickness for up to 3 h, using calipers. The columns and points are the mean ± SD *n* = 3 (panels **A** and **B**) and 4 in (panel **C**); * *p* < 0.05 compared to PBS or the corresponding control; *** *p* < 0.001 compares to PBS or the corresponding control.

The mean of clot formation time in plasma incubated with BaCol PLA_2_ was 446 ± 31 s, while plasma incubated with PBS (control) clotted after 208 ± 30 s (*n* = 4 each; *p* = 0.0007) The indirect hemolytic activity (Figure 6A) and cleavage of the synthetic substrate 4-nitro-3-octanoyloxy-benzoic acid (4-NOBA) (Figure 6B) by BaCol PLA_2_ was equal to the complete venom using 15 µg/µL of purified toxin that generated a 26 mm halo and 15 µg/µL of complete venom that caused a 20 mm halo; the same cleavage capacity of 4-NOBA was also evidenced for both. BaCol PLA_2_ caused mouse footpad edema, with 5 µg and 20 µg increasing the paw thickness by 45 ± 0.17% and 60 ± 0.11%, respectively, 1 h after toxin inoculation. Maximum edema was observed after 2 h (Figure 6C; *p* < 0.0001 compared to the control).

**Figure 7:**
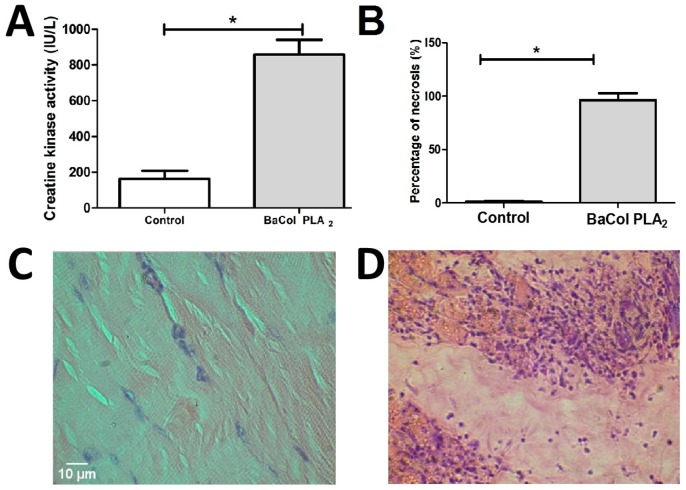
Myonecrosis caused by BaCol PLA_2_ in mouse gastrocnemius muscle. (**A**) Plasma CK activity 3 h after injection of BaCol PLA_2_ (50 µg/100 µL, i.m.) compared to mice injected i.m. with 0.9% NaCl (control); (**B**) Percentage of necrosis determined as described in the Methods; (**C**,**D**) Histology of gastrocnemius muscle injected with 0.9% NaCl (control) and BaCol PLA_2_, respectively. Hematoxylin and Eosin staining. The columns in (**A**,**B**) are the mean ± SD (*n* = 4); * *p* < 0.05 compared to control mice. The white bar represents 10 microns.

BaCol PLA_2_ induced moderate myonecrosis, seen as an increase in plasma creatine kinase (CK) activity 3 h after toxin injection (50 µg/mouse) compared to the control group (Figure 7A). Histopathological analysis confirmed the occurrence of tissue damage (Figure 7B). Compared to control muscle (Figure 7C) where discrete infiltration of neutrophils, mild vascular congestion and absence of necrosis was observed, BACol PLA_2_ caused severe, diffuse muscle fiber damage with vacuolization and necrosis of the sarcoplasm (Figure 7D).

**Figure 8:**
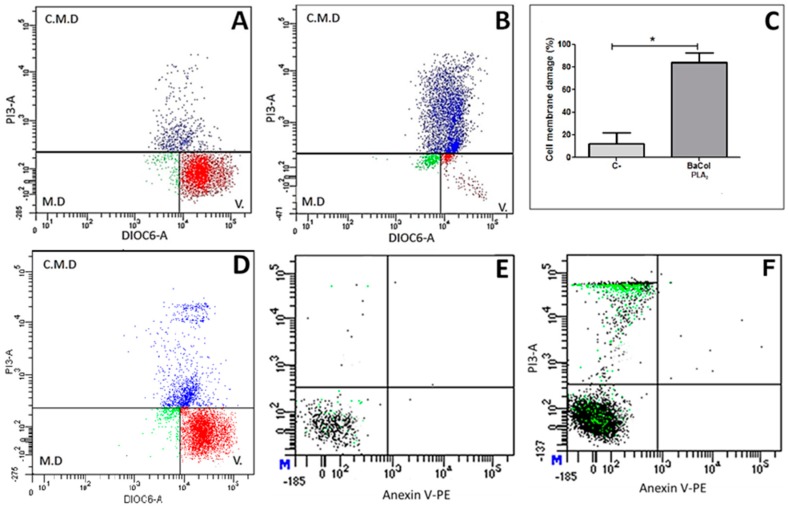
Cytotoxicity of BaCol PLA_2_ in U937 lymphocytes. (**A**) Normal cells not incubated with BaCol PLA_2_ (viability: 85%); (**B**) Cells incubated with BaCol PLA_2_ (1.6 µg/µL) (membrane damage: 82%); (**C**) Cell membrane damage (%) induced by BaCol PLA_2_ compared to control (non-treated) cells. The columns are the mean ± SD (*n* = 3); (**D**) Cell membrane damage induced by BaCol PLA_2_ (0.16 µg/µL); (**E**) Negative control for annexin V conjugated with phycoerythrin; (**F**) Cells incubated with BaCol PLA_2_ (0.16 µg/µL). There was no phosphatidylserine exposure indicative of early apoptosis. * *p* < 0.05 compared to control cells.

Incubation of U937 cells with BaCol PLA_2_ (1.6 µg/µL) resulted in extensive (82%) damage to the cytoplasmic membrane, seen as high intensity fluorescence for PI (Figure 8B,C). The corresponding damage in control cells was 15% (Figure 8A,C), with most of them showing high intensity fluorescence for DIOC_6_ and negative fluorescence for PI, indicating that they were still viable. At a BaCol PLA_2_ concentration of 0.16 µg/µL, the plasma membrane damage was ~22% (Figure 8D,F); at this concentration, staining with a phytoerythrin-annexin V conjugate detected no phosphatidylserine externalization, indicating there was no early apoptosis. This finding suggested that the mechanism of cell death caused by BaCol PLA_2_ was more compatible with necrosis (Figure 8D). Figure 8E shows the annexin V control without BaCol PLA_2_.

## 3. Discussion

Many acidic PLA_2_ isolated from snake venoms are devoid of pharmacological activities or toxicity, including myotoxicity [11,14,15]. In this report, we describe the structural and functional characteristics of BaCol PLA_2_, a new acidic myotoxic Asp49 PLA_2_ isolated from Colombian *B. asper* venom. The toxin was obtained in a single chromatographic step by RP-HPLC and had a molecular mass of 14,180.69 Da and pI of 4.4. The complete sequence obtained from *B. asper* venom gland cDNA indicated that this protein differed from other acidic PLA_2_ previously reported for this species [11]. BaCol PLA_2_ contained Asp at position 48 (position 49 is the catalytic diad, according to the numbering of Renetseder et al. [13]), and was therefore classified as an Asp49 PLA_2_. We used homology modeling to obtain a hypothetical 3D structure of BaCol PLA_2_. The model obtained was reliable since the stereochemical quality, the compatibility of the 3D structure with the amino acid sequence and the energy profile of the generated model were similar of those described for other acidic PLA_2_s [14]. In addition, our model showed the conserved residues involved in calcium binding (Tyr28, Gly30, Gly32, His48 and Asp49) and in the catalytic network (His48, Asp99, Tyr52, and Tyr73) [13]. In common with other snake venom acidic PLA_2_ [7,12], BaCol PLA_2_ had highly conserved amino acids, such as 14 cysteines involved in the formation of seven disulfide bonds.

BaCol PLA_2_ was myotoxic, as shown by an increase in circulating creatine kinase and histological damage (involving fiber contraction, clumping and degeneration) to gastrocnemius muscle fibers. However, myotoxicity is not a consistent characteristic of acidic PLA_2_ isolated from *Bothrops* venoms. Thus, whereas acidic PLA_2_ such as BinTX-I from *B. insularis* [12], Bp-PLA_2_ from *B. pauloensis* [16] and BmooTX-I from *B. moojeni* [17] venoms are myotoxic, others, such as PLA_2_s from *B. jararaca* [18], Costa Rican *B. asper* [11], *B. pirajai* [14] and *B. moojeni* [19] are not. BaCol PLA_2_ showed high identity (91%) with Costa Rican BaspPLA_2_-II; the incomplete identity suggests that variations in some amino acid residues could play an important role in determining the occurrence of myonecrosis.

Similar results were found with BmooTX-I obtained from *B. moojeni*, in which extensive cellular destruction and abundant leukocitary infiltrate were described and, further displaying contracted and clumped fibers in different stages of degeneration [17]. Bp-PLA_2_ from *B. pauloensis* [16], whose myotoxicity was confirmed by the increase in the CK activity was similar to induced by whole venom. In the same way, BinTX-I from *B. insularis*, also increased CK activity. Nevertheless, morphological analysis, showed BinTX-I produced less damaged proportion of fibers than the venom [12].

BaCol PLA_2_ was highly edematogenic, with the peak response occurring 2 h after toxin injection. This finding agrees with other acidic PLA_2_ with which BaCol PLA_2_ shares considerable identity [10,11,12,14,19].

BaCol PLA_2_ was cytotoxic to U937 lymphocytes at concentrations ranging from 0.016 µg/µL (data not shown) to 1.6 µg/µL after 24 h. Santos Filho et al. [17] reported that BmooTX-I was able to hydrolyze cell membrane phosphatidylcholine to release free fatty acids and lysophospholipids, resulting in cellular damage. In contrast, Bl-PLA_2_ from *Bothrops leucurus* did not affect the viability of human peripheral blood mononuclear cells [20]. Similarly, BaspPLA_2_-II did not damage the cell membrane of C2C12 skeletal muscle myoblasts in culture [11]. Staining of U937 lymphocytes with annexin V and PI revealed no early apoptosis (no exposure of phosphatidylserine), indicating that cell death was by necrosis rather than apoptosis. Mora et al. [21] reported that a basic Lys49 PLA_2_ from Costa Rican *B. asper* induced apoptosis at concentrations of 5–25 µg/mL, whereas necrosis was observed at a concentration of 50 µg/mL. In contrast, our findings revealed no marked differences in the effects seen with two concentrations of BaCol PLA_2_ (0.16 μg/μL and 1.6 µg/μL).

BaCol PLA_2_ showed anticoagulant activity based on prolongation of the plasma clotting time. Anticoagulant activity has not generally been evaluated among other acidic PLA_2_, except for BaPLA_2_-II, which was found to have no such activity [11]. Compared to the strong anticoagulant activity of basic PLA_2_s, that of BaCol PLA_2_ was quite weak. This discrepancy could be explained by the finding that in PLA_2_s with high anticoagulant activity the putative anticoagulant site located between residues 54 and 77 is positively charged, contrary to PLA_2_ with moderate or low anticoagulant activity, in which there is a predominance of negative or neutral charges in this region [22]. This explanation based on structure agrees with the presence of four aspartic acid and two glutamic acid in this segment in the amino acid sequence of BaCol PLA_2_. In Table 1 can be observed the comparative characteristics of BaCol PLA_2_ and other acidic PLA_2_ describe before.

In Colombia there are few studies for *B. asper* acidic myotoxic PLA_2_ and therefore it is necessary to deepen in order to understand better its action mechanism and its relationship with the effects induced by venom.

## 4. Materials and Methods

### 4.1. Venom

*Bothrops asper* venom from central Magdalena in Antioquia was donated by the Antioquia University serpentarium. The pool of venom was obtained by manual milking of 30 adult specimens. The venom was centrifuged, lyophilized and frozen at −70 °C until used.

### 4.2. Animals

Male Swiss-Webster mice were supplied by the Animal House (Sede de Investigacion Universitaria–SIU) of the Universidad de Antioquia and maintained under standard conditions with access to food and water *ad libitum*. The experimental protocol was approved by the institutional Committee for the Use and Care of Research Animals at the Universidad de Antioquia (license nos. 70 (2011) and 102 (2016)).

### 4.3. Isolation of BaCol PLA_2_

Venom (10 mg) was dissolved in 200 µL of 0.1% trifluoroacetic acid (TFA; solvent A), centrifugated and applied to a reverse-phase high performance liquid chromatography (RP-HPLC) C18 semipreparative column (250 × 10 mm, 5 μm particle; Restek, Bellefonte, PA, USA), using a Shimadzu Prominence-20A chromatograph. Elution was performed at 2 mL/min by applying solution B (acetonitrile, containing 0.1% TFA) as follows: 5% B for 5 min, 15% B over 15 min, 45% over 75 min, 70% B over 85 min, 90 min 70%. The elution profile was monitored at 215 nm in a UV/VIS photodiode array detector (Shimadzu, Kyoto, Japan). The peaks were collected and evaluated by SDS PAGE on a 12% polyacrylamide gel and peaks with electrophoretic bands of ~15 kDa were subsequently screened for hemolytic activity. Of the peaks obtained, only two had hemolysis halo greater than 20 mm, but only one was located around 15 kDa and that is the reason to choose it. The enzymatically active fraction was subsequently applied to a liquid chromatography (RP-HPLC) C18 analytical column (250 × 4.6 mm; Restek, Bellefonte, PA, USA), using a Shimadzu Prominence-20A chromatograph. Elution was performed at 1 mL/min by applying solution B (acetonitrile, containing 0.1% TFA) as follows: 0% B for 0 min, 100% B over 30 min. The elution profile was monitored at 215 nm in a UV/VIS photodiode array detector (Shimadzu, Kyoto, Japan).

The electrophoretic homogeneity of BaCol PLA_2_ was evaluated by SDS-PAGE. For this, 20 µg of protein was loaded onto a 12% polyacrylamide gel and run in a Mini-Protean Tetra^®^ electrophoresis system (Bio-Rad, Hercules, CA, USA) at 150 V. The gel was subsequently stained with Coomassie blue *R*-250 [23].

### 4.4. Molecular Mass and N-Terminal Determination

Protein purity and molecular mass were also examined by ESI-Q-ToF mass spectrometry using a MicrQ-TOF III mass spectrometer (Bruker Daltonics, Billerica, MA, USA) coupled to an LC-20AT liquid chromatograph (Shimadzu, Kyoto, Japan). Two mobile phases were used: water (A) and acetonitrile (B), both in the presence of 0.1% (*v*/*v*) formic acid. Chromatographic runs were done on a C18 reverse-phase column (4.5 mm × 100 mm, 1.8 μm). The elution conditions were consisted of a 0–85% linear gradient of solvent B for 60 min at a flow rate of 0.2 mL/min. The sample column and automatic applicator were held at 25 °C and 10 °C, respectively. The mass spectrometer was operated at 4.5 kV with a solvation temperature of 180 °C, positive mode with an ionization interval between 100 m/z and 3000 m/z, a nitrogen flow of 6 L/min and pressure of 0.8 bars. The data were processed using Bruker Data Analysis software (version 3.3, Billerica, MA, USA, 2011).

The *N*-terminal sequence was determined using a Shimadzu automatic protein sequencer (PPSQ-23A model). An aliquot of the sample (~1 mg/mL) was applied to the sequencer and the sequence was determined by Edman degradation [24]. The *N*-terminal sequence was subsequently aligned with other snake venom PLA_2_s using BLAST [25] and MultAlin [26] programs.

### 4.5. cDNA and Nucleotide Sequencing

A venomous gland from a dead adult specimen of *B. asper* from the serpentarium of the Universidad de Antioquia was used to obtain the complete sequence of the toxin. The gland total RNA was extracted with QIAzol^®^ according to the manufacturer’s instructions (Qiagen, Hilden, Germany). The RNA was subsequently retrotranscribed using Superscript III enzyme (Invitrogen^®^) as recommended by the manufacturer (Invitrogen Corporation, Carlsbad, CA, USA). The mRNA was transformed into cDNA using a dNTP mix and specifically designed *N*-terminal-based primers: external primer 5′-GTTTGGCCAGATGATGAGCG-3′ and internal primer 5′-GGCGATGATCCGTGCAAAAA-3′. The cDNA was cloned into the vector pGEM-*T* Easy (Promega, Madison, WI, USA) and DH5-*α E. coli* were transformed. PCR was used to detect the presence of the vector with the toxin sequence. The plasmid was obtained from these transformed colonies using the construct and a QIA prep^®^ spin miniprep kit (Qiagen, Hilden, Germany). The product of this extraction was sequenced (Macrogen, Seoul, Korea) to certify that it corresponded to whole plasmids with the gene inserted in the multiple cloning site. Sequencing was done from the vector T7 and SP6 promoters. The DNA sequence data were analyzed and translated into the amino acid sequence using Mega 6 software [27]. Protein sequence homology in the Swiss-Prot database was searched using FASTA3 [28] and sequence alignments were generated with CLUSTAL W 2.1. The theoretical isoelectric point was calculated with ExPASy [29].

### 4.6. Bidimensional Electrophoresis and Determination of Isoelectric Point

For the first dimension, 30 µg of BaCol PLA_2_ was applied to a 7 cm long immobilized pH gradients strip (pH 3–10). The strip was rehydrated overnight at room temperature in 125 µL of rehydration solution containing 8 M urea, 2% (*w*/*v*) CHAPS, 1% of immobilized pH gradient (IPG) buffer solution, 19 nM dithiothreitol (DTT) and bromophenol blue. Isoelectric focusing was done in three steps: first step—500 V for 30 min, second step—1000 V for 30 min, and third step—5000 V for 2 h. The strip was subsequently equilibrated for 20 min in 19 mM DTT, 50 mM Tris, 6 M urea, 30% glycerol (*v*/*v*) and 2% SDS (*w*/*v*) followed by a further 20 min in the same solution, except that DTT was replaced by 0.2 M iodioacetamide. For the second dimension, the isoelectric focusing strip was place on top of a 10% polyacrylamide gel (10 × 10 cm, 1.5 mm thick). Electrophoresis was done at room temperature in two steps (10 mA per gel for 15 min and then 20 mA per gel for 1 h) and the gel then stained with Coomassie Brilliant Blue. The biodimensional gel was digitized using an ImageScanner III scanner (GE Healthcare Life Sciences, Issaquah, WA, USA) in transmission mode and the image was analyzed using Image Master 2D Platinum v 7.05 (GE Healthcare, Little Chalfont, UK) software to determine the isoelectric point.

### 4.7. Molecular Modeling

The NCBI Basic Local Alignment Search Tool (BLAST) for sequence similarities was used to search the crystal structures of the closest homologs available in the Brookhaven Protein Data Bank (PDB). The results retrieved by NCBI BLAST identified chain *X* of PLA_2_ from *Bothrops jararacussu* (PDB ID: 1WMV_X) with a resolution of 1.79 Å as a suitable template with an identity score of 78%, an E value of 7 × 10^−67^ and coverage of 100%. The three-dimensional model of BaCol PLA_2_s was obtained using the program Modeller (9.17) [30,31]. This program is completely automated and capable of generating energy minimized protein models by satisfying spatial restraints on bond distances and dihedral angles extracted from the template PDB file. Modeller performs automatic loop modeling and model optimization. Numerous runs of Modeller were used to generate the most plausible model. The stereochemical excellence of the protein structure and overall structural geometry were confirmed using Procheck [32]. The energy of residues was checked with ProSA using the web service ProSA-web [33,34]. Verify 3D software was used to determine the compatibility of an atomic model (3D) with its own amino acid sequence (1D) by assigning a structural class based on its location and environment (*α*, *β*, loop, polar, non-polar, etc.), as well as by comparing the results with good database structures [35].

### 4.8. PLA_2_ Activity

PLA_2_ activity was evaluated in vitro by indirect hemolysis on agar gel containing human erythrocytes and egg yolk [36] and by cleavage of the synthetic substrate 4-NOBA [37,38], the latter modified for 96-well plates. For the latter assay, the standard assay mixture contained 200 μL of buffer (10 mM Tris-HCl, 10 mM CaCl_2_, 100 mM NaCl, pH 8.0), 20 μL of 4-NOBA, 20 μL of water and 20 μL of PLA_2_ (1 μg/μL) in a final volume of 260 μL. After adding BaCol PLA_2_ (20 μg), the mixture was incubated at 37 °C for 60 min after which the absorbance was determined at 405 nm using a multi-well plate reader (Awareness, Stat Fax 3200, Westport, CT, USA). The activity was expressed as the increase in absorbance/min during the linear phase of the reaction. All the assays were done in triplicate. *Bothrops asper* venom and synthetic substrate 4-NOBA without toxin were used as positive and negative controls, respectively.

### 4.9. Edematogenic Activity

BaCol PLA_2_ (5 μg and 20 μg) diluted in 50 μL of phosphate-buffered saline (PBS) was injected subcutaneously in the right hind paw footpad and control mice were injected with 50 μL of PBS (*n* = 4 mice/group, experiment was performed in duplicate). Footpad thickness was measured (in mm) with calipers 1, 2 and 3 h after toxin injection. Hind paw footpad thickness was measured prior to the inoculations and edema was expressed as the increase of this measure in each of the three moments with respect to the initial measurement [39].

### 4.10. Myotoxicity

Mice were inoculated in the right gastrocnemius muscle with 0.9% NaCl (100 µL; control; *n* = 4) or BaCol PLA_2_ (50 µg in 100 µL of 0.9% NaCl; *n* = 4). After 3 h, a 70 µL blood sample was collected from the tail vein into heparinized capillaries. The plasma was separated and used to quantify CK activity (CK-NAC UV, Wiener Lab^®^, Rosario, Argentina) according to the manufacturer’s instructions, with the final absorbance being measured at 280 nm [40,41]. The gastrocnemius muscle was dissected and fragments were processed for histopathological analysis. Sections 4 µm thick were stained with hematoxylin and eosin and examined for myonecrosis, edema, congestion and leukocyte infiltrate. The extent of myonecrosis was calculated by examining 3 non-overlapping regions in each cross-sections/muscle or mouse (4 mice/group) as the percentage of the examined area corresponding to necrotic fibers, (myofibril disorganization, delta lesions, vacuolation).

### 4.11. Anticoagulant Activity

The effect of BaCol PLA_2_ on the clotting time of human plasma was examined by preincubating 20 μg of enzyme (in 50 μL of PBS) with 500 μL of human citrated plasma for 10 min at 37 °C, in duplicate. Subsequently, 100 μL of 0.25 M CaCl_2_ was added and time to clot (in seconds) was recorded manually. Plasma aliquots preincubated with PBS were used as controls [36]. The assay was performed in triplicate.

### 4.12. Cell Viability and Cell Death

U937 cells were used to assess alterations on the mitochondrial permeability transition (MPT) and damage to the cell membrane. The cells were suspended in RPMI 1640 medium supplemented with 10% fetal bovine serum and then plated at a density of 300.000 cells/well (total volume/well: 300 μL) followed by incubation at 37 °C for 24 h. DIOC_6_ and propidium iodide (PI) were used to determine the MPT and cell membrane damage, respectively, by flow cytometry (FACSCanto II). BaCol PLA_2_ cytotoxicity was evaluated at concentrations ranging from 0.016 μg/μL to 1.6 μg/μL after 24 h. The cells that showed high intensity fluorescence for DIOC_6_ and negative fluorescence for PI were classified as viable, whereas those with high intensity fluorescence for PI were considered to be dead (as a consequence of damage to the cytoplasmic membrane). To determine the type of cell death induced by PLA_2_, cells were suspended in medium containing PI and annexin V (Invitrogen^®^, Carlsbad, CA, USA). Cells were considered apoptotic when they showed positive staining for annexin V and negative staining for PI; non-apoptotic cells showed no staining for either dye. Cells in the early stages of apoptosis were positive for annexin V and negative for PI, whereas necrotic cells showed double-positive staining. Cells suspended in RPMI 1640 and stains without PLA_2_, were used as negative controls in both experiments.

### 4.13. Statistical Analysis

Numerical data were expressed as the mean ± SD. Statistical comparisons were done using Student’s *t*-test or one-way ANOVA followed by the Bonferroni test, with *p* < 0.05 indicating significance. All data analyses were done using the software GraphPad Prism 5.01 version for Windows (GraphPad Software, San Diego, CA, USA, 2007).

## Figures and Tables

**Table 1:** Comparative characteristics between BaCol PLA_2_ and other acidic PLA_2_s.

Reference	Phospholipase	Isoelectric Point	Myotoxic Activity	Edematogenic Activity	Platelet Aggregation	Anti-Clotting Activity
Posada et al, 2017	BaCol PLA_2_	4.4	Yes	Yes	-	Moderate
Cogo et al., 2006	BinTx-I	5.05	Yes	Yes	-	-
Rodrigues et al., 2007	Bp-PLA_2_	4.3	Yes	Yes	Inhibits	-
Santos et al., 2008	BmooTx-I	4.2	Yes	Yes	Inhibits	-
Fernandez et al., 2010	Basp PLA_2_-II	4.9	No	Yes	Does not inhibit	No
Teixeira et al., 2011	Bpir PLA_2_-I	4.8	No	Yes	Inhibits	-
Silveira et al., 2013	Bmoo PLA_2_	5.2	No	Yes	Inhibits	Yes
AndriaoEscarso et al., 2002	BthA-I-PLA_2_	4.5	No	Yes	Inhibits	Low
Nunes et al., 2011	Bl PLA_2_	5.4	Low	Low	-	-
Serrano et al., 1999	BJ PLA_2_	-	-	No	Inhibits	-

## Supplementary Materials

The following are available online at www.mdpi.com/2072-6651/9/11/342/s1, Figure S1: Overlay of the proposed structure of BaCol PLA_2_ (Cyan) with the template structure (PDB ID: 1UMV_X, Purple). Green sphere represents a Ca^2+^ ion; Figure S2: Ramachandran plot of modeled BaCol PLA_2_. The favored and most favored region is red and brown respectively. Yellow is the generally allowed and disallowed regions is pale yellow; Figure S3: Verify-3D analysis. Green-dashed line represents the limit score of 0.2. Positive scores suggest that the residues are compatible with their environments in the model build forBaCol PLA_2_. The lowest and the highest values are shown; Table S1: Residues with a score under 0.2; Figure S4: ProSA energy plot calculated for the BaCol PLA_2_ homology model. The energy plot displayed byProSA shows the local model quality by plotting energies in function of the amino acid sequence position. Positive values correspond to problematic or erroneous parts of a model. When the fragment of 10 residues was evaluated, most of them were in the negative region. However, when a fragment of 40 residues was evaluated none of the residues is outside of the negative region.
