# Supplementary material for: Isolation and Functional Characterization of an Acidic Myotoxic Phospholipase A2 from Colombian Bothrops asper Venom
Source: Toxins (Basel). 2017 Oct 26;9(11):342. doi: 10.3390/toxins9110342 (PMC5705957; doi:10.3390/toxins9110342)
Supplement: Supplementary file 1 [file toxins-09-00342-s001.pdf]

SupplementaryMaterials: Isolation and functional characterization of an acidic myotoxic phospholipase A2 from Colombian *Bothrops asper* venom

Silvia Posada Arias, Paola Rey-Suárez, Andrés Pereáñez J, Cristian Acosta, Mauricio Rojas, Lucilene Delazari dos Santos, Rui Seabra Ferreira Jr and Vitelbina Núñez

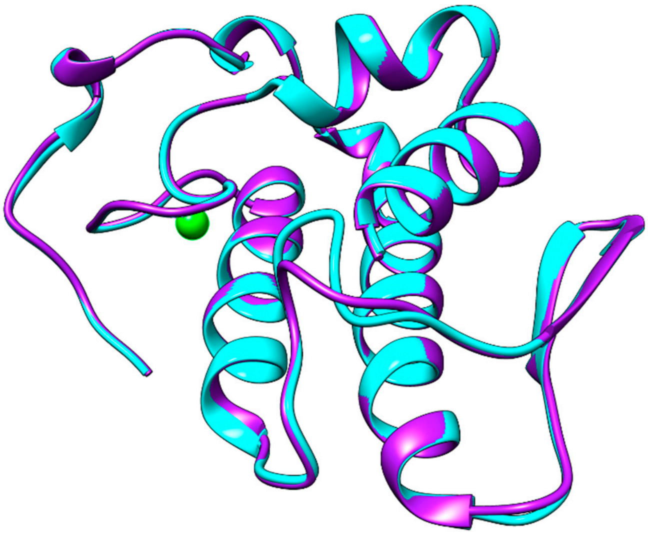

Figure S1. Overlay of the proposed structure of BaCol PLA2 (Cyan) with the template structure (PDB ID: 1UMV_X, Purple). Green sphere represents a Ca^{2+} ion.

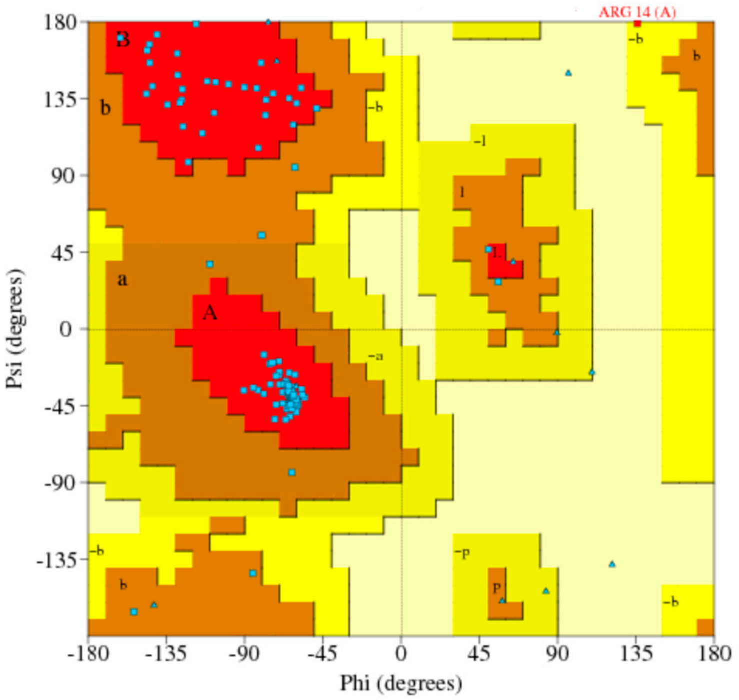

Figure S2. Ramachandran plot of modeled BaCol PLA2. The favored and most favored region is red and brown respectively. Yellow is the generally allowed and disallowed regions is pale yellow.

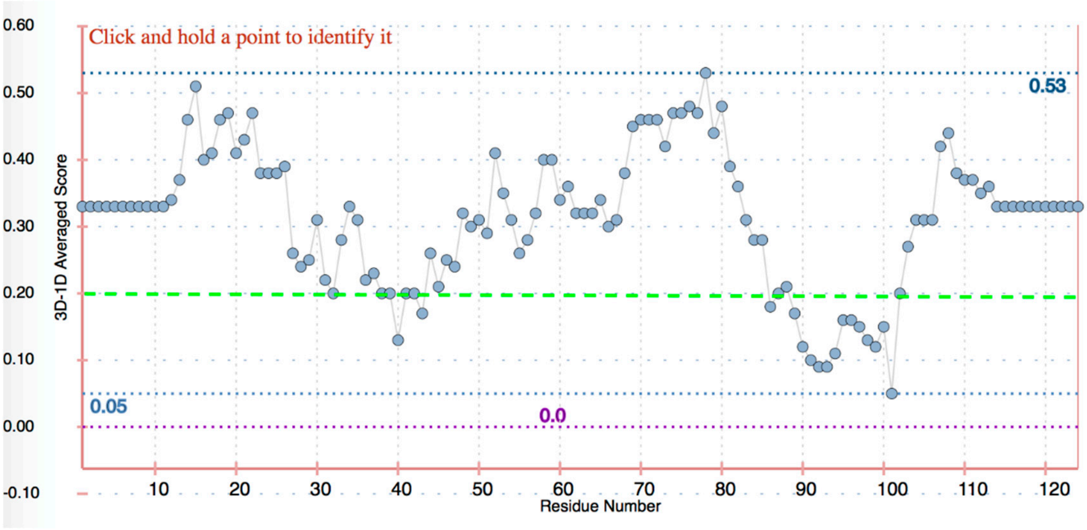

Figure S3. Verify-3D analysis. Green-dashed line represents the limit score of 0.2. Positive scores suggest that the residues are compatible with their environments in the model build forBaCol PLA_2. The lowest and the highest values are shown.

Table S1. Residues with a score under 0.2.

Residues	3D-1D	Average Scores
avg C	43	0.17
avg E	89	0.17
avg C	90	0.12
avg D	91	0.10
avg R	92	0.09
avg V	93	0.09
avg A	94	0.11
avg A	95	0.16
avg I	96	0.16
avg C	97	0.15
avg F	98	0.13
avg R	99	0.12
avg D	100	0.15
avg N	101	0.05

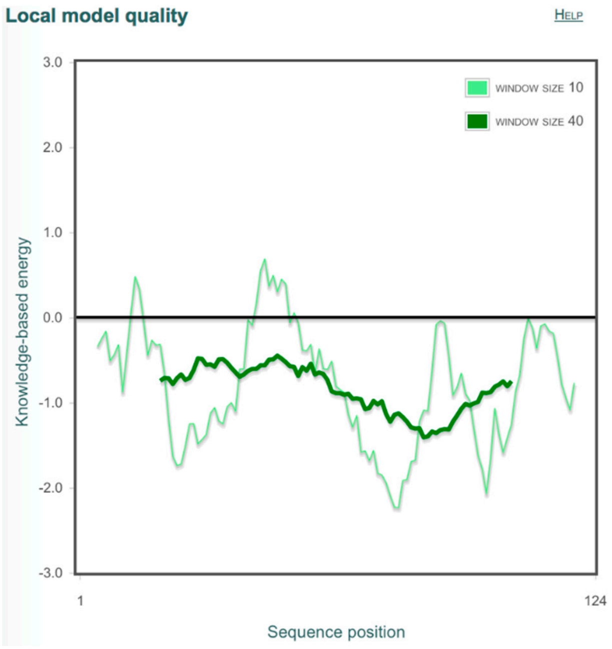

Figure S4. ProSA energy plot calculated for the BaCol PLA_2 homology model. The energy plot displayed byProSA shows the local model quality by plotting energies in function of the amino acid sequence position. Positive values correspond to problematic or erroneous parts of a model. When the fragment of 10 residues was evaluated, most of them were in the negative region. However, when a fragment of 40 residues was evaluated none of the residues is outside of the negative region.
